# Supplementary material for: Investigation of the Efficacy of Benzylidene-3-methyl-2-thioxothiazolidin-4-one Analogs with Antioxidant Activities on the Inhibition of Mushroom and Mammal Tyrosinases
Source: Molecules. 2024 Jun 18;29(12):2887. doi: 10.3390/molecules29122887 (PMC11206987; doi:10.3390/molecules29122887)
Supplement: Supplementary file 1 [file molecules-29-02887-s001.zip › molecules-3004402-supplementary.pdf]

Supporting Information

For

Investigation of the efficacy of benzylidene-3-methyl-2-thioxothiazolidin-4-one analogs with antioxidant activities on the inhibition of mushroom and mammal tyrosinases

Hye Jin Kim [1,†], Hee Jin Jung [1,†], Young Eun Kim [1], Daeun Jeong [1], Hyeon Seo Park [1], Hye Soo Park [1], Dongwan Kang [2], Yujin Park [2], Pusoon Chun [3], Hae Young Chung [4] and Hyung Ryong Moon [1,*]

[1] Department of Manufacturing Pharmacy, College of Pharmacy and Research Institute for Drug Development, Pusan National University, Busan 46241, Republic of Korea; khj3358@pusan.ac.kr (H.J.K.); hjjung2046@pusan.ac.kr (H.J.J.); k87115@pusan.ac.kr (Y.E.K.); 202487128j@pusan.ac.kr (D.J.); gustj6956@pusan.ac.kr (H.S.P.); hyesoo0713@pusan.ac.kr (H.S.P.)

[2] Department of Medicinal Chemistry, New Drug Development Center, Daegu-Gyeongbuk Medical Innovation Foundation, Daegu 41061, Republic of Korea; kdw4106@kmedihub.re.kr (D.K.); pyj1016@kmedihub.re.kr (Y.P.)

[3] College of Pharmacy and Inje Institute of Pharmaceutical Sciences and Research, Inje University, Gimhae 50834, Gyeongnam, Republic of Korea; pusoon@inje.ac.kr

[4] Department of Pharmacy, College of Pharmacy, Pusan National University, Busan 46241, Republic of Korea; hyjung@pusan.ac.kr

* Correspondence: mhr108@pusan.ac.kr; Tel.: +82-51-510-2815; Fax: +82-51-513-6754

† These authors contributed equally to this work.

Contents

Figure S1. ^{1}H NMR spectrum of analog **1**……..……..…………………………………….....3

Figure S2. ^{13}C NMR spectrum of analog **1**……..……..…………..………..…………..…4

Figure S3. ^{1}H NMR spectrum of analog **2**………..…………………..………………..…5

Figure S4. ^{13}C NMR spectrum of analog **2**………..……………………….....…………..6

Figure S5. ^{1}H NMR spectrum of analog **3**……..………………...….……………….…......7

Figure S6. ^{1}H-coupled NMR spectrum of analog **3**……..…………………..…..…………8

Figure S7. ^{1}H NMR spectrum of analog **4**……………....………………………………….9

Figure S8. ^{13}C NMR spectrum of analog **4**……..……………………………………..………10

Figure S9. ^{1}H NMR spectrum of analog **5**……………..………………….………....……..11

Figure S10. ^{13}C NMR spectrum of analog **5**………………..…………………..……….....12

Figure S11. ^{1}H NMR spectrum of analog **6**……………..…………………..…..……….13

Figure S12. ^{13}C NMR spectrum of analog **6**……………...……………...…………….. …..14

Figure S13. ^{1}H NMR spectrum of analog **7**……………...……………..……………..….15

Figure S14. ^{13}C NMR spectrum of analog **7**.………………..…….…………….…..…..16

Figure S15. ^{1}H NMR spectrum of analog **8**……………..…………………………………17

Figure S16. ^{13}C NMR spectrum of analog **8**………………..……….…..…………........18

Figure S17. HRMS spectrum of analog **1**…………………………………………………….19

Figure S18. HRMS spectrum of analog **3**…………………………………………………….20

Figure S19. HRMS spectrum of analog 5……………………………………………………..21

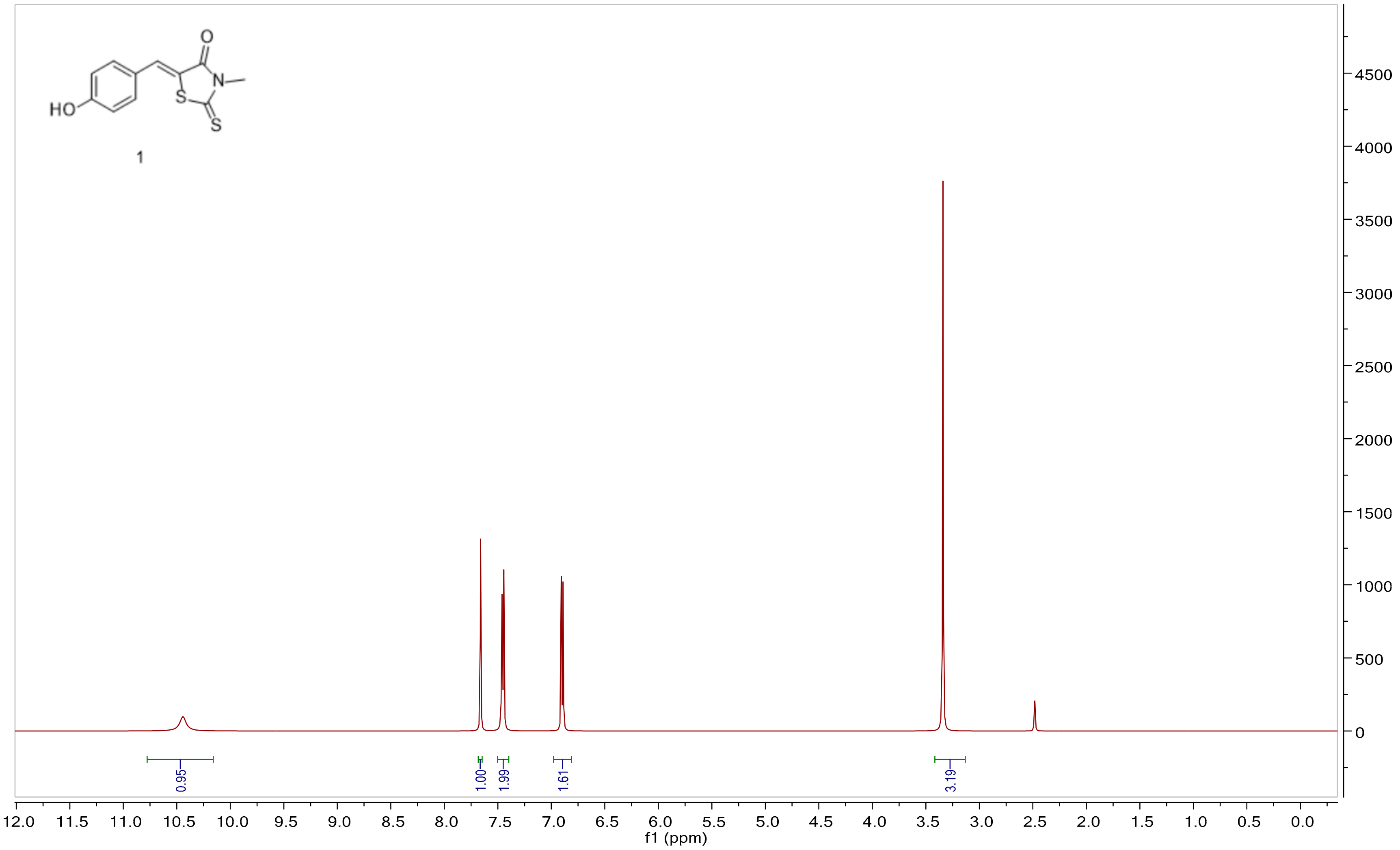

Figure S1. ^{1}H NMR spectrum of analog **1**

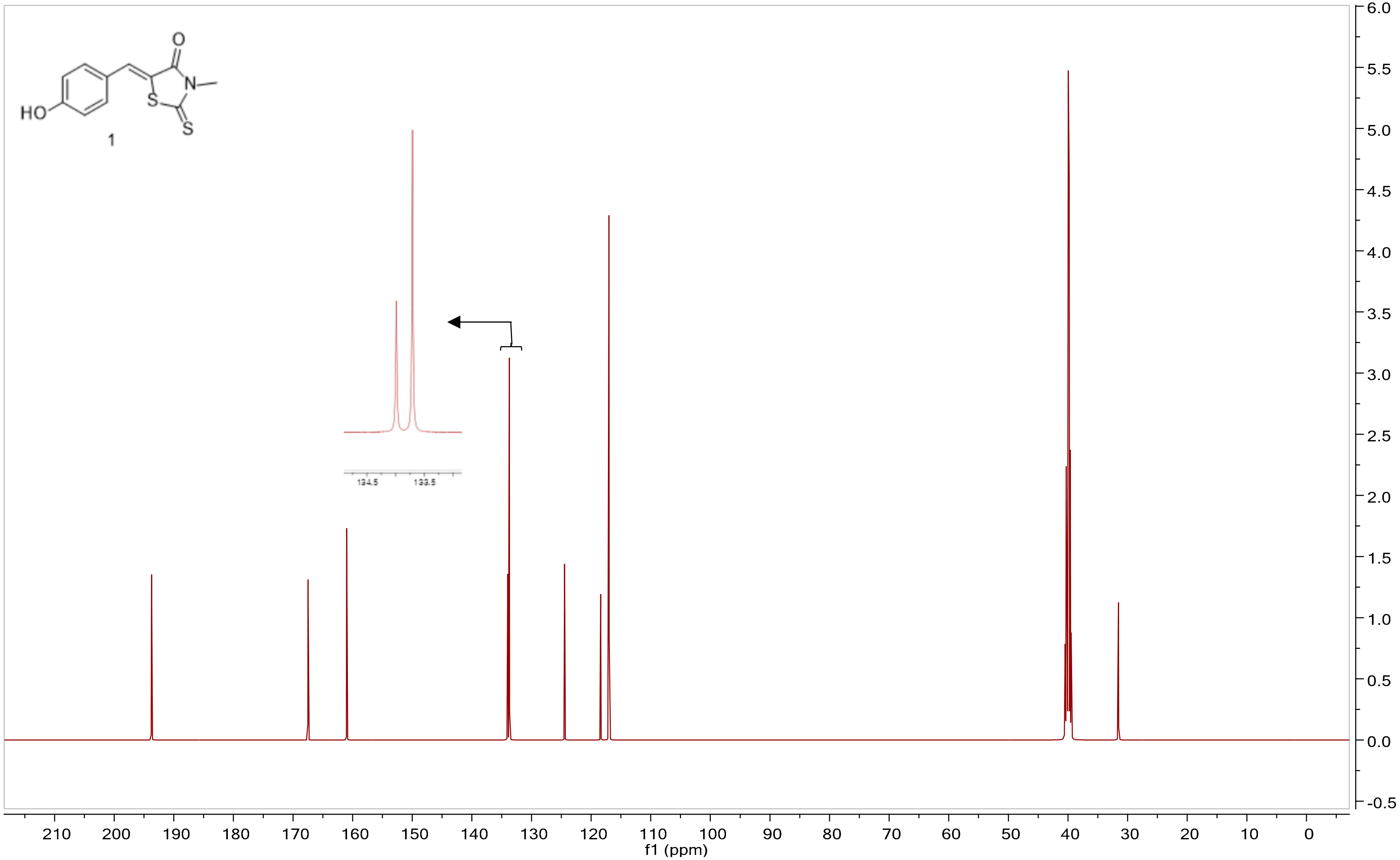

Figure S2. ^{13}C NMR spectrum of analog **1**

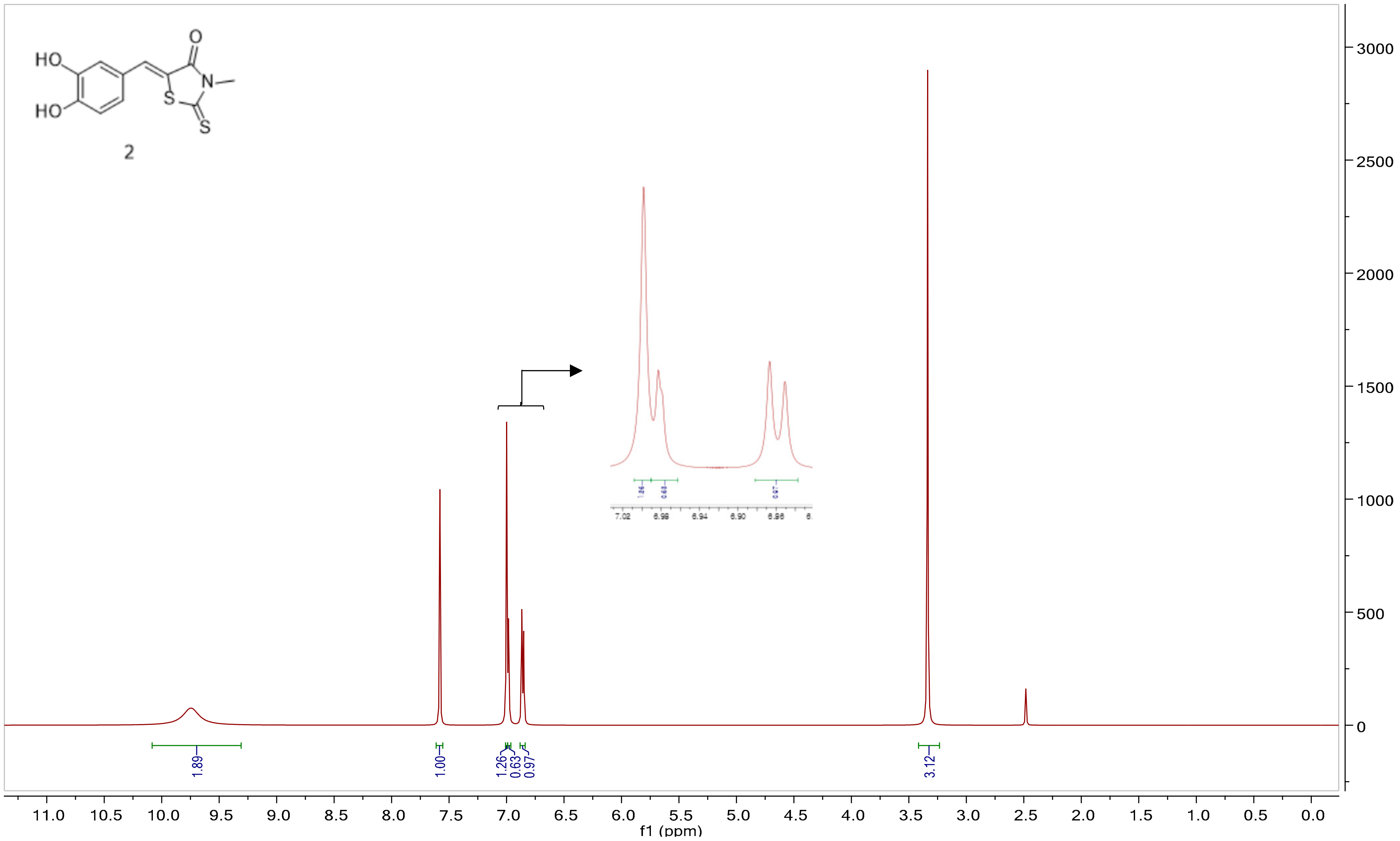

Figure S3. ^{1}H NMR spectrum of analog **2**

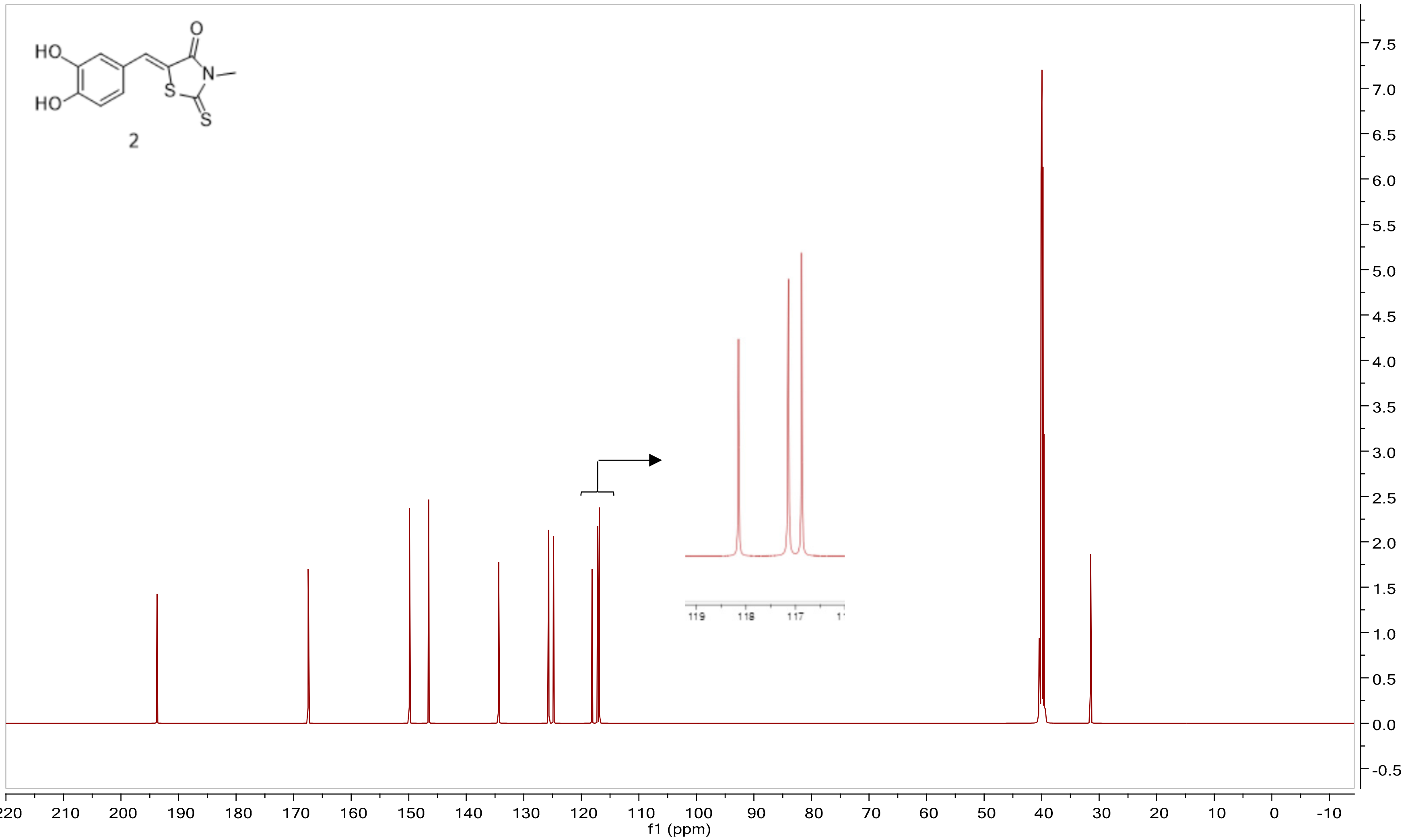

Figure S4. ^{13}C NMR spectrum of analog **2**

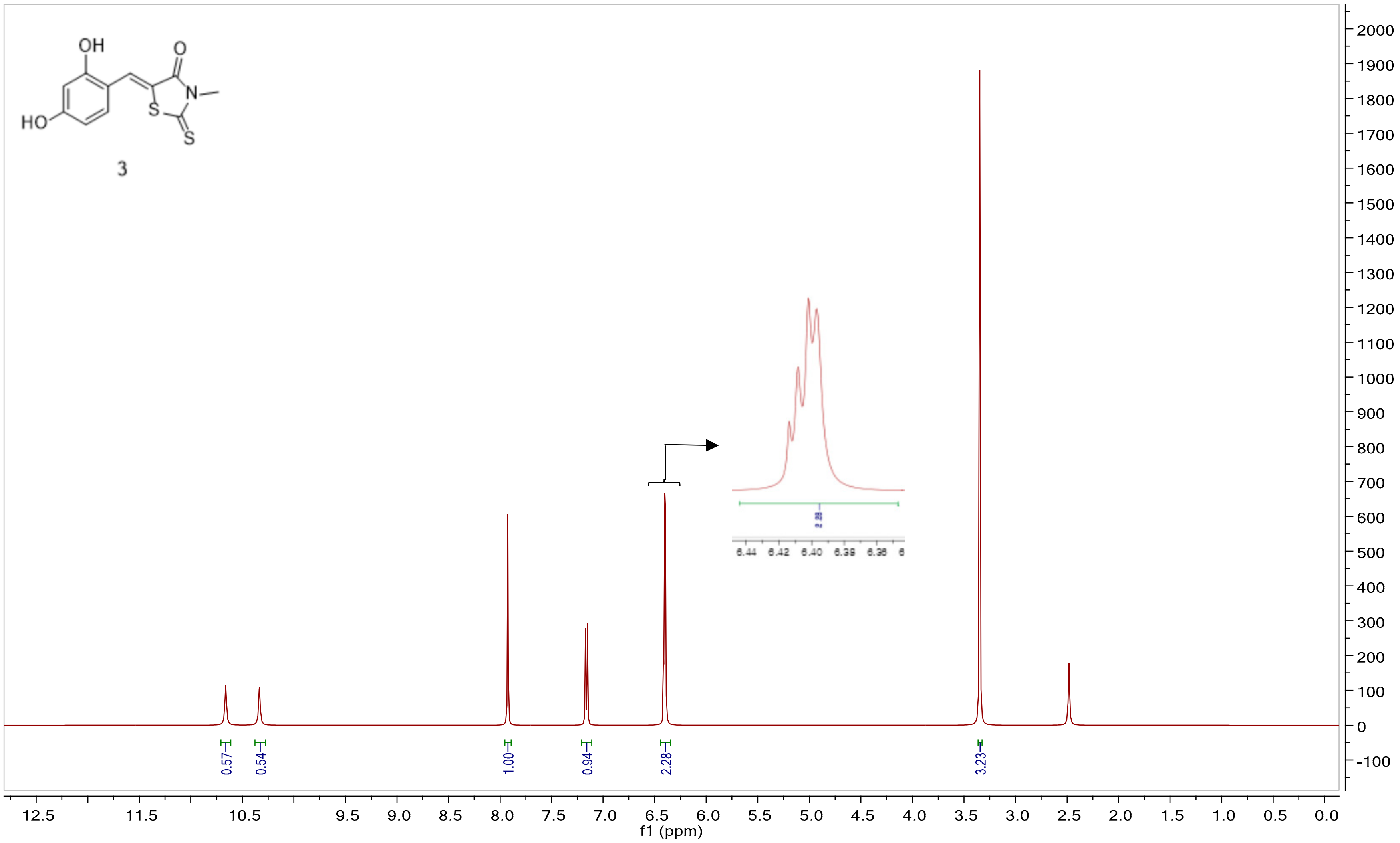

Figure S5. ^{1}H NMR spectrum of analog **3**

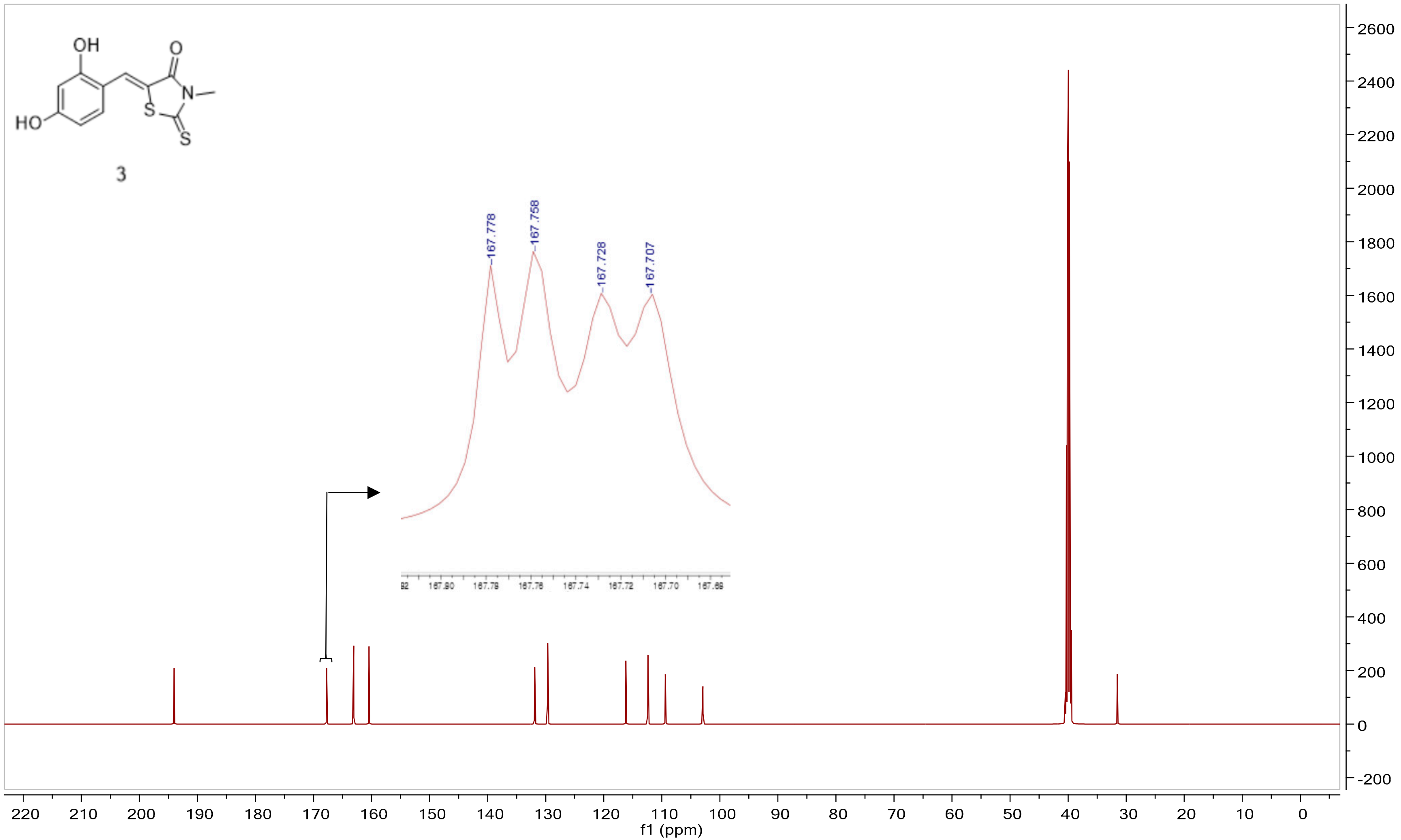

Figure S6. ^{1}H-coupled NMR spectrum of analog **3**

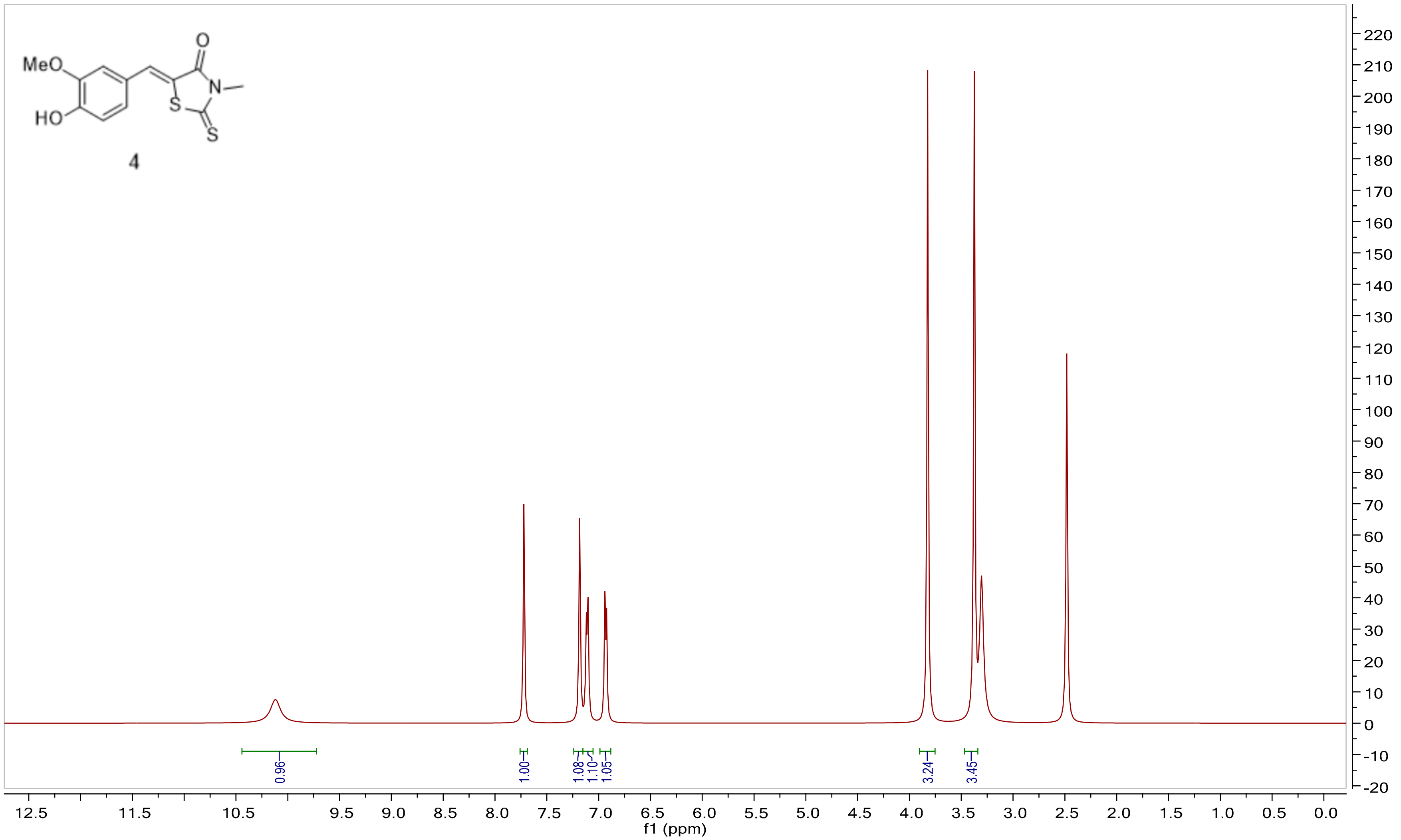

Figure S7. ^{1}H NMR spectrum of analog **4**

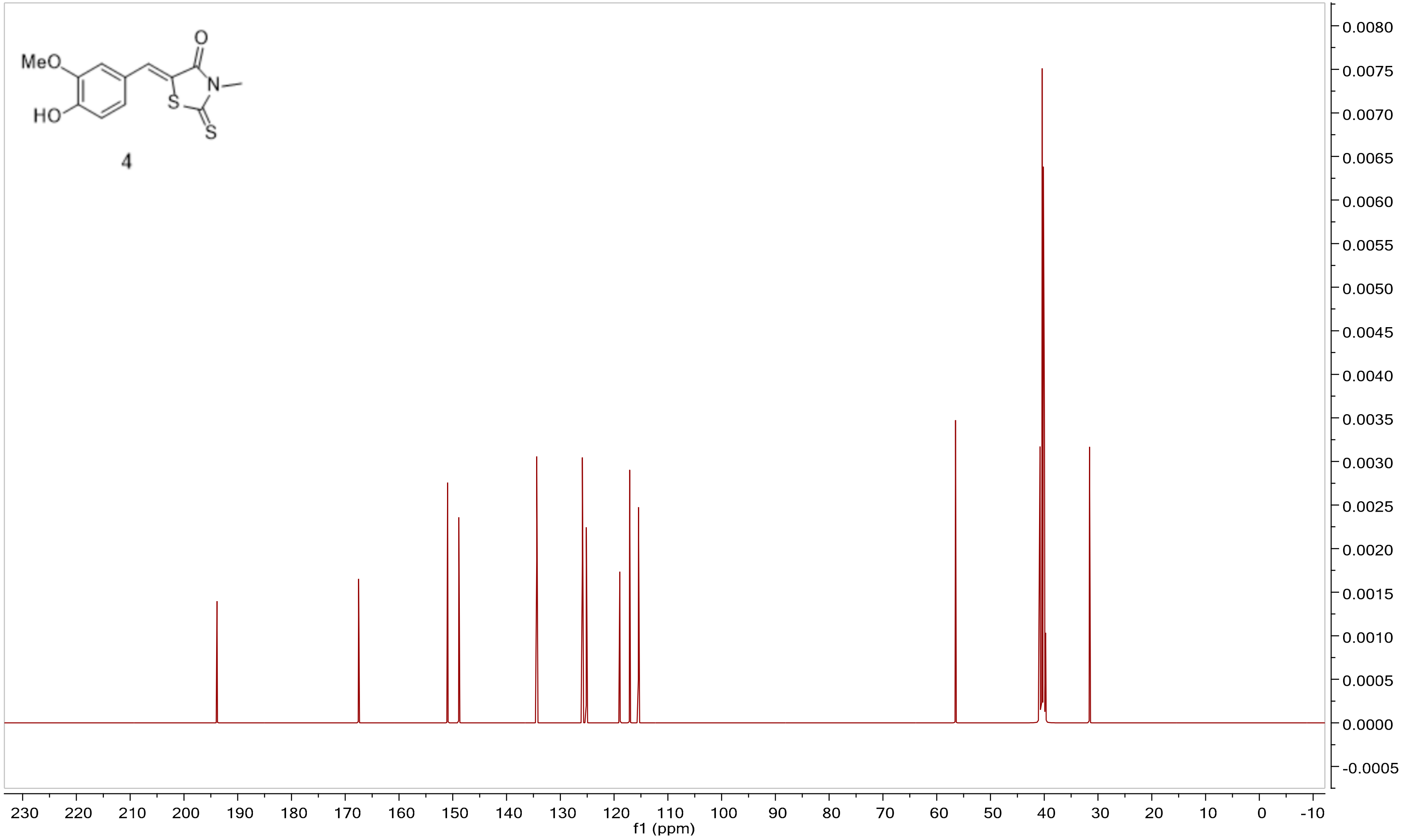

Figure S8. ^{13}C NMR spectrum of analog **4**

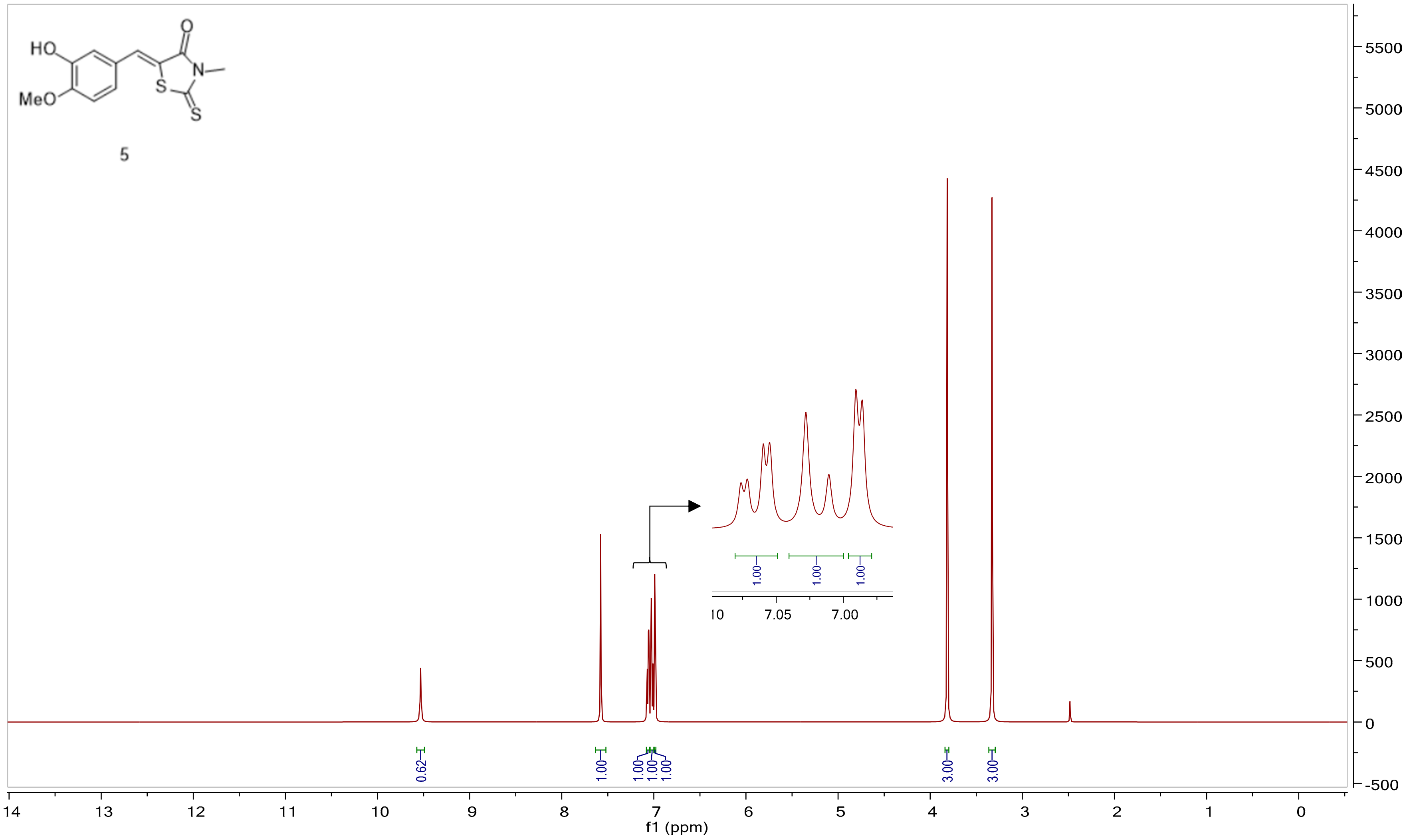

Figure S9. ^{1}H NMR spectrum of analog **5**

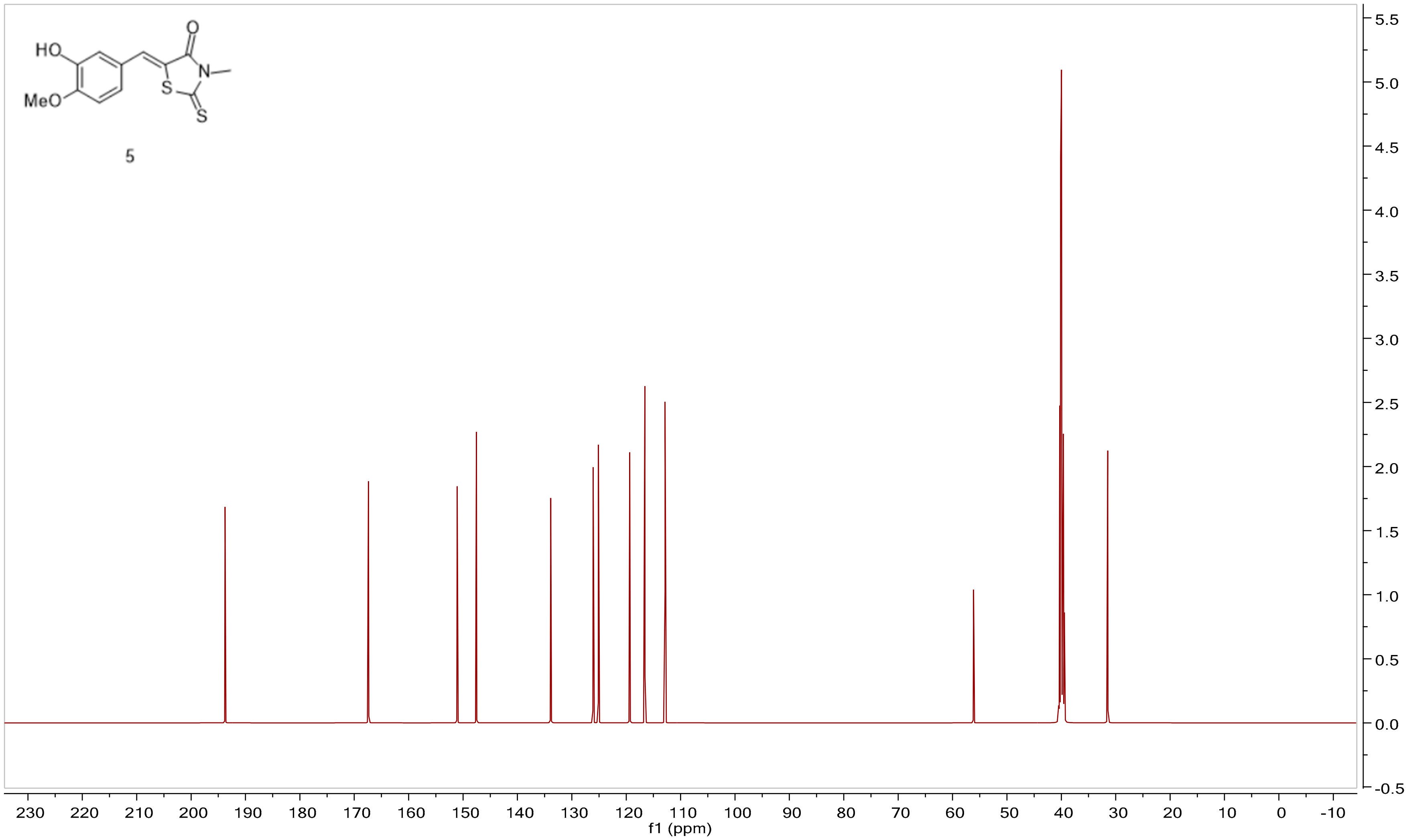

Figure S10. ^{13}C NMR spectrum of analog **5**

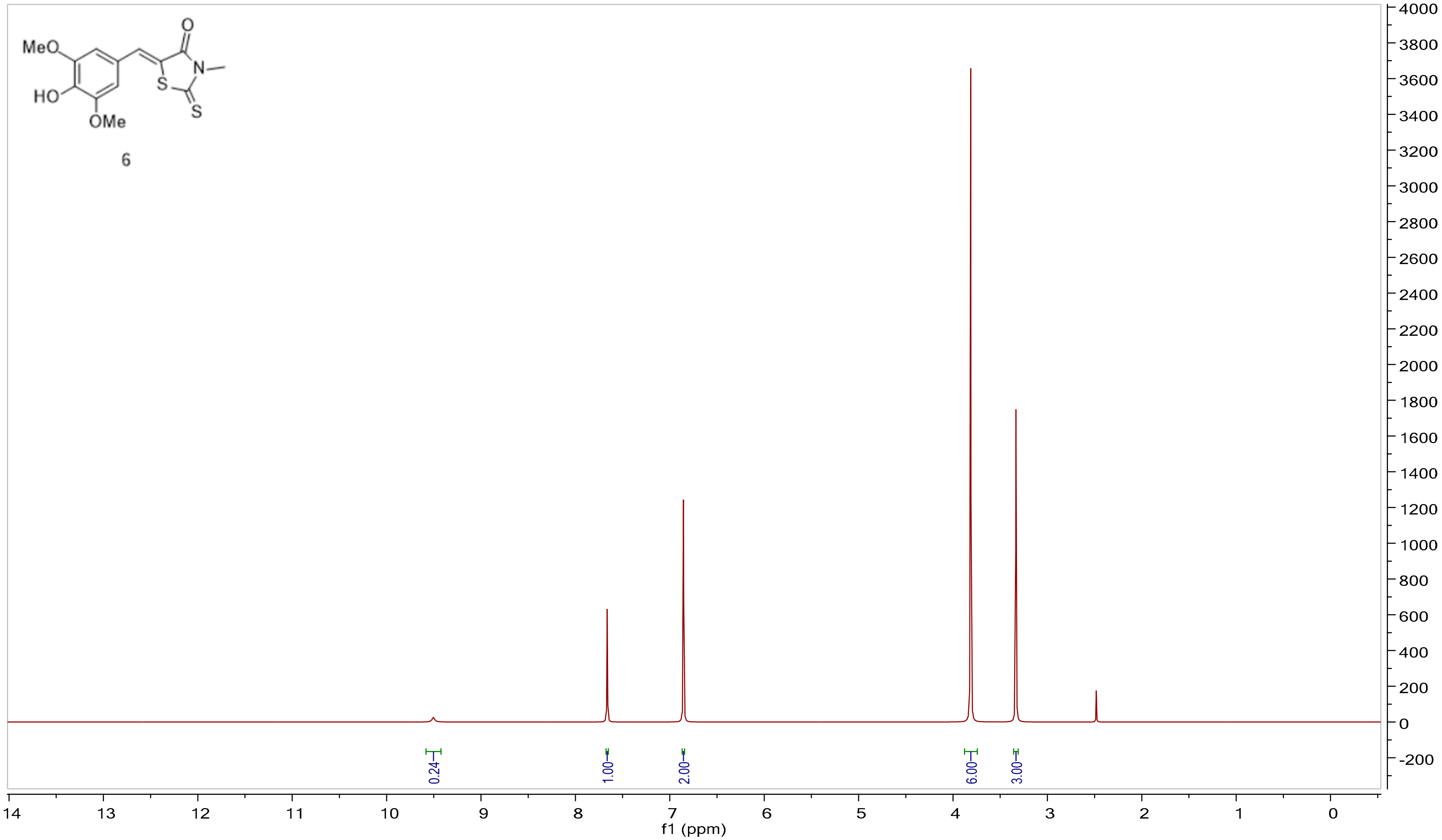

Figure S11. ^{1}H NMR spectrum of analog **6**

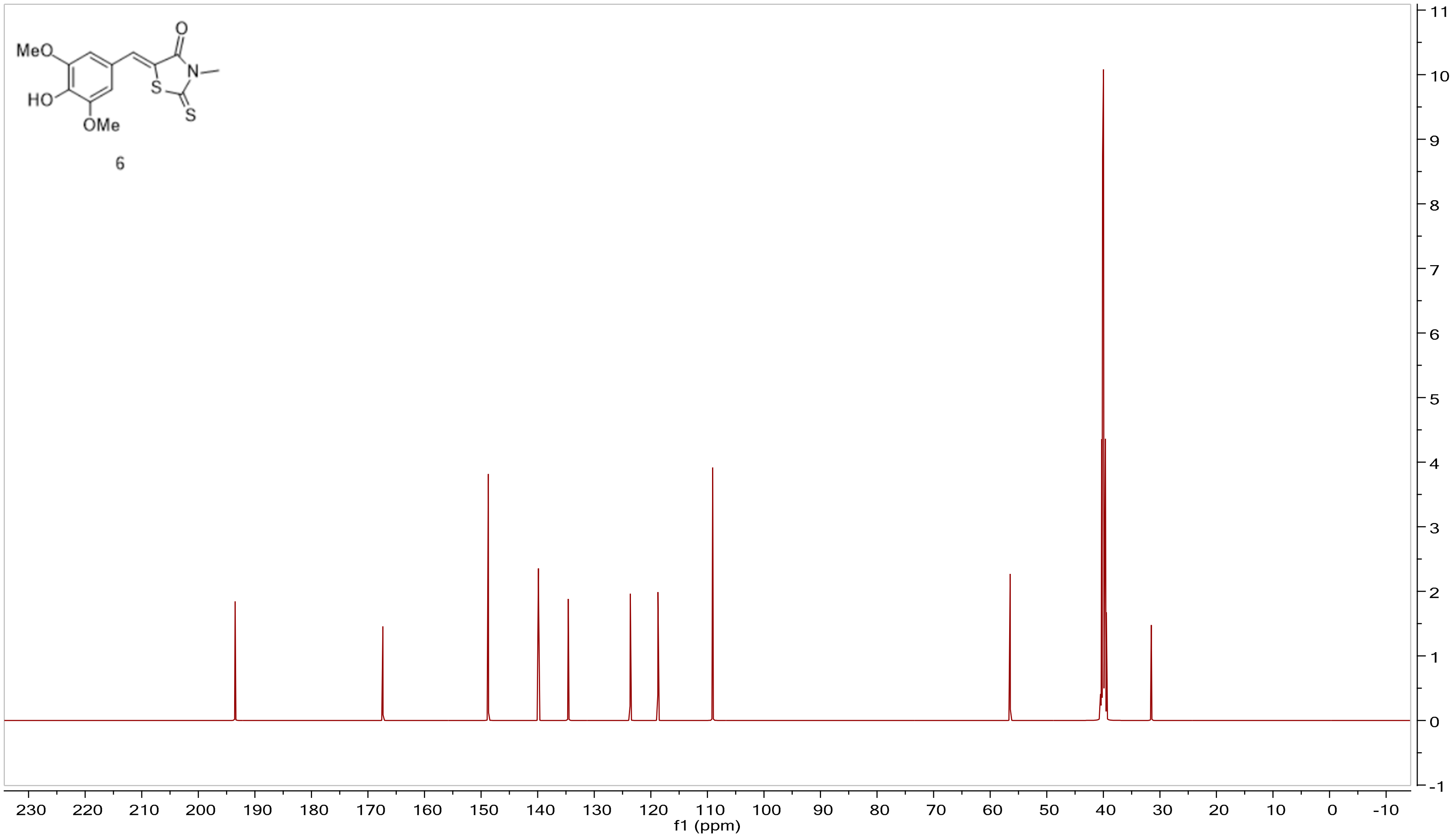

Figure S12. ^{13}C NMR spectrum of analog **6**

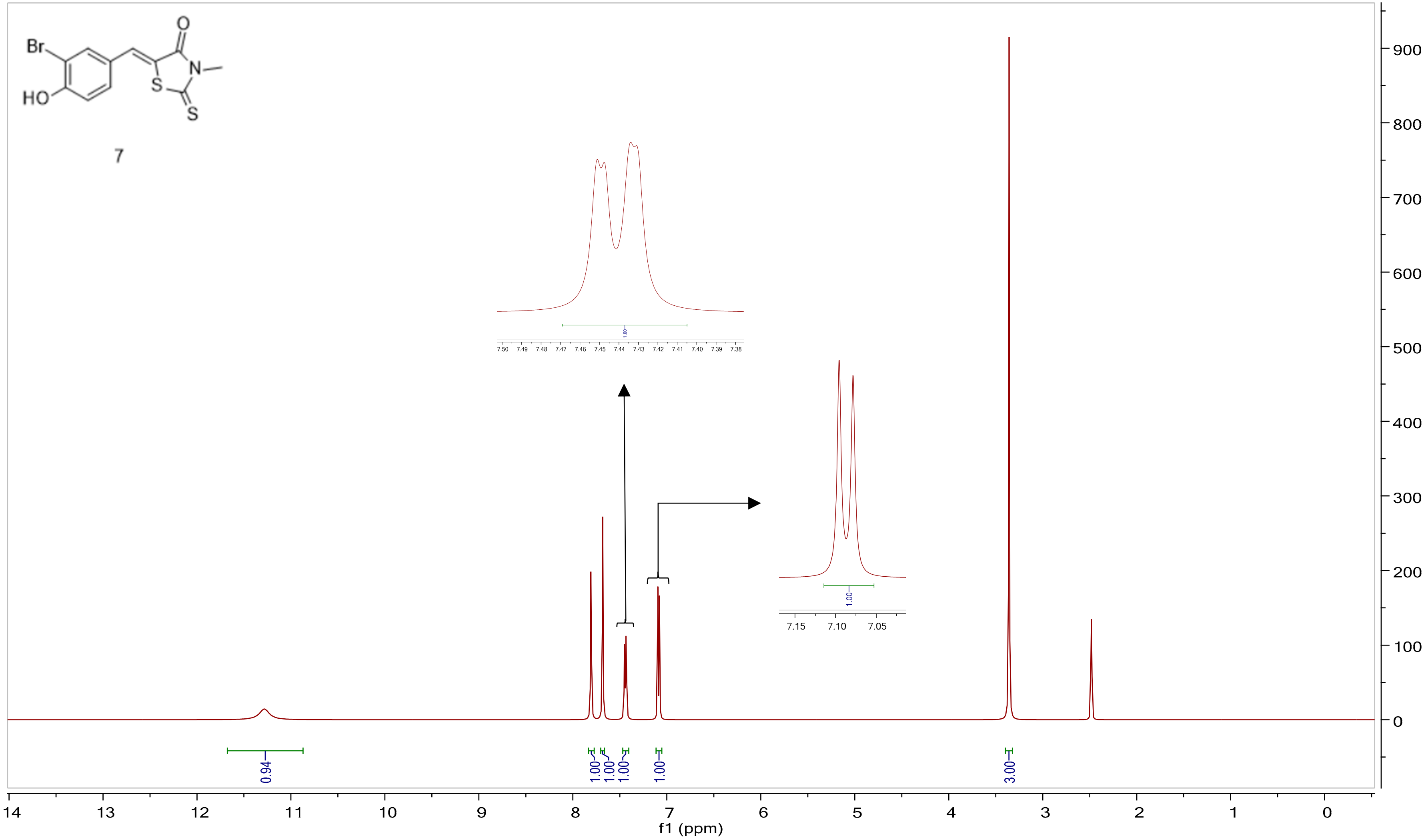

Figure S13. ^{1}H NMR spectrum of analog **7**

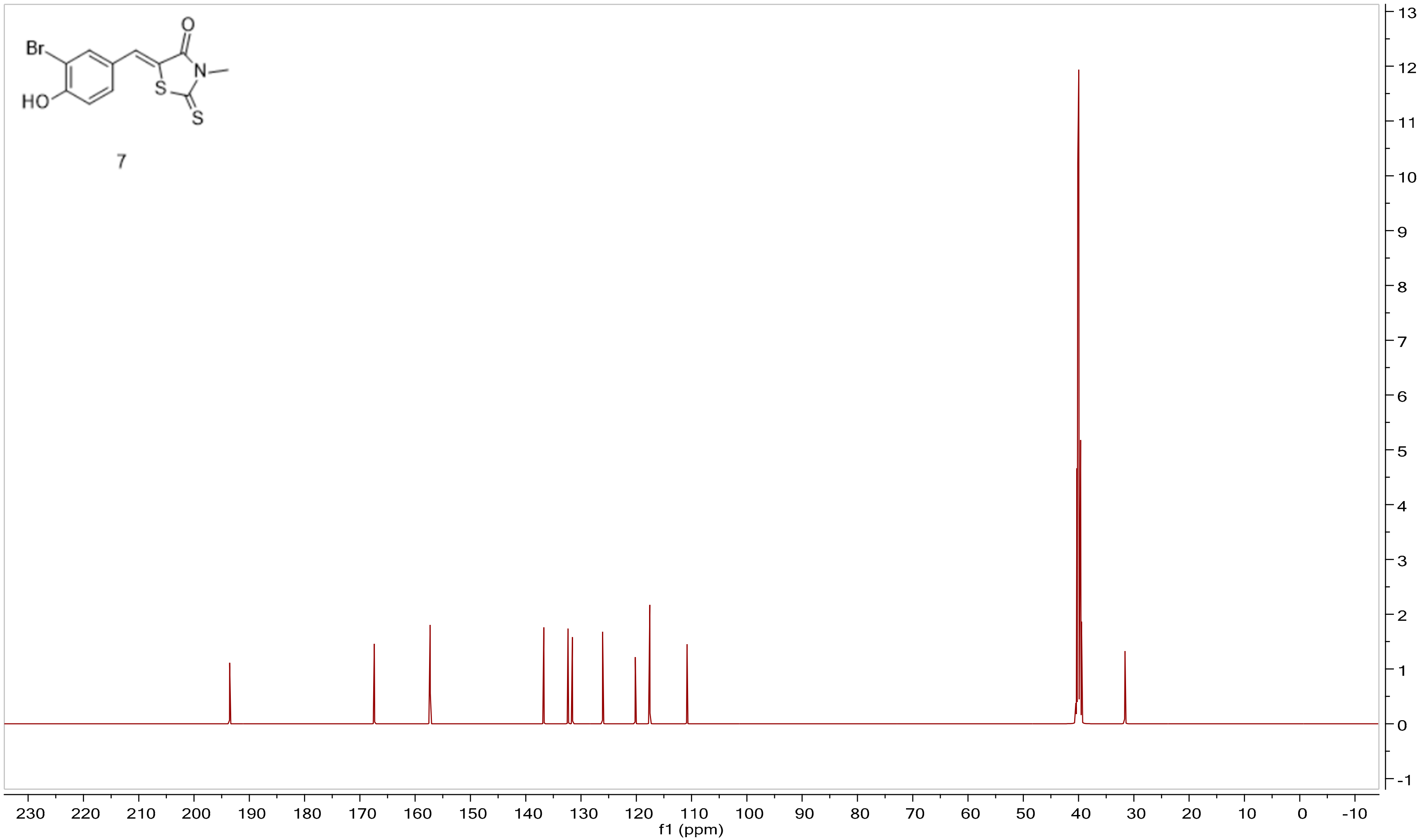

Figure S14. ^{13}C NMR spectrum of analog **7**

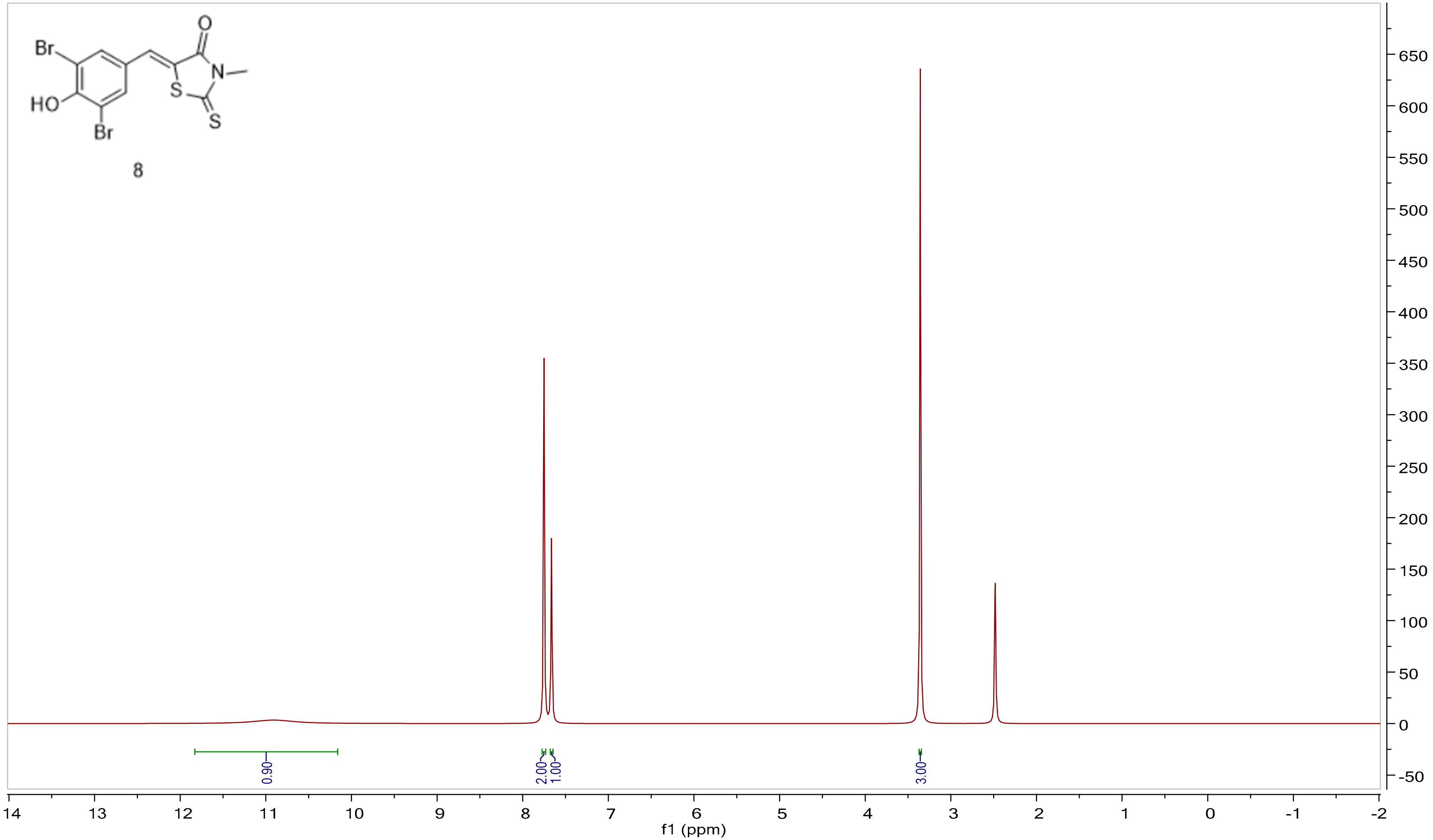

Figure S15. ^{1}H NMR spectrum of analog **8**

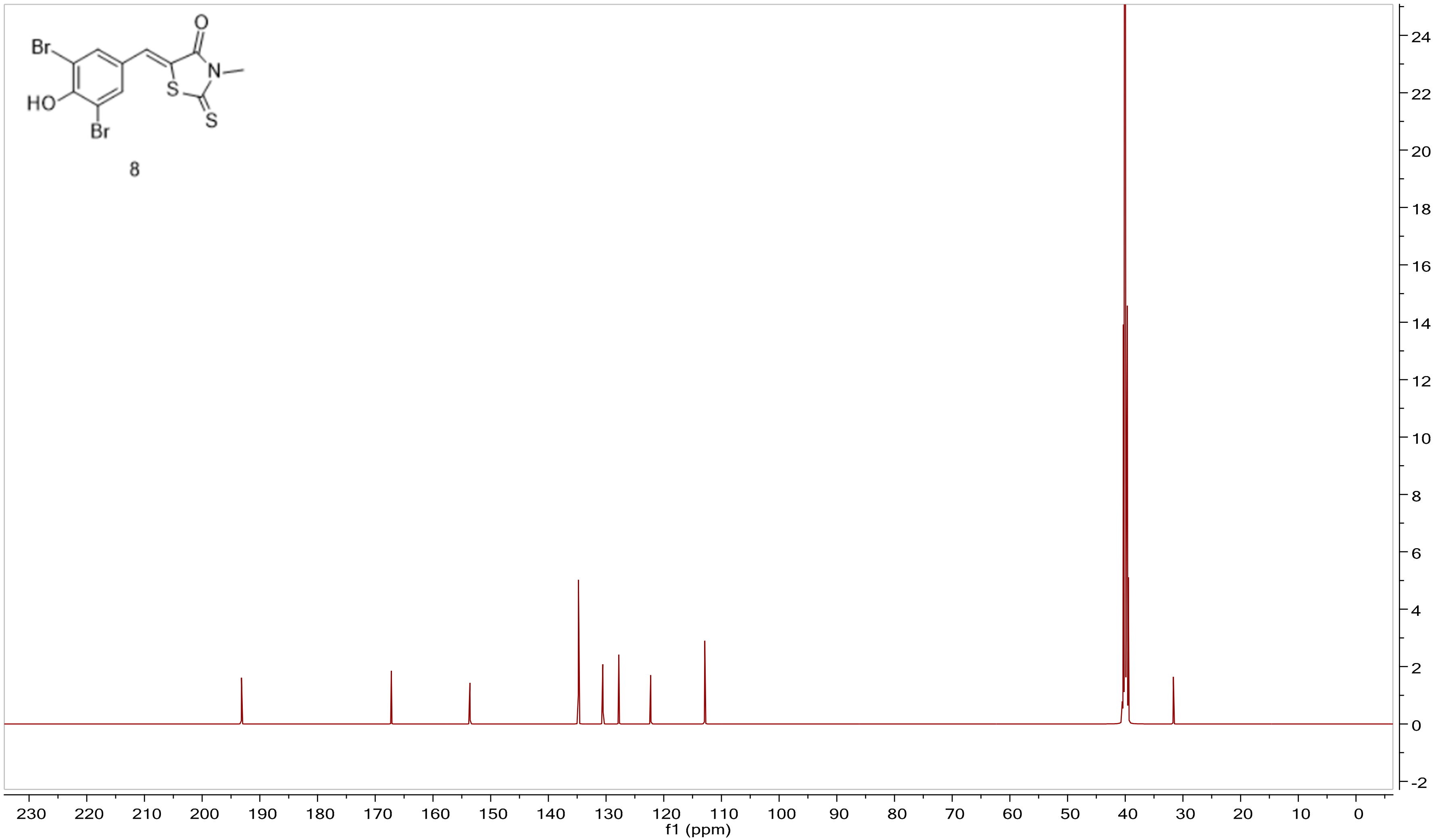

Figure S16. ^{13}C NMR spectrum of analog **8**

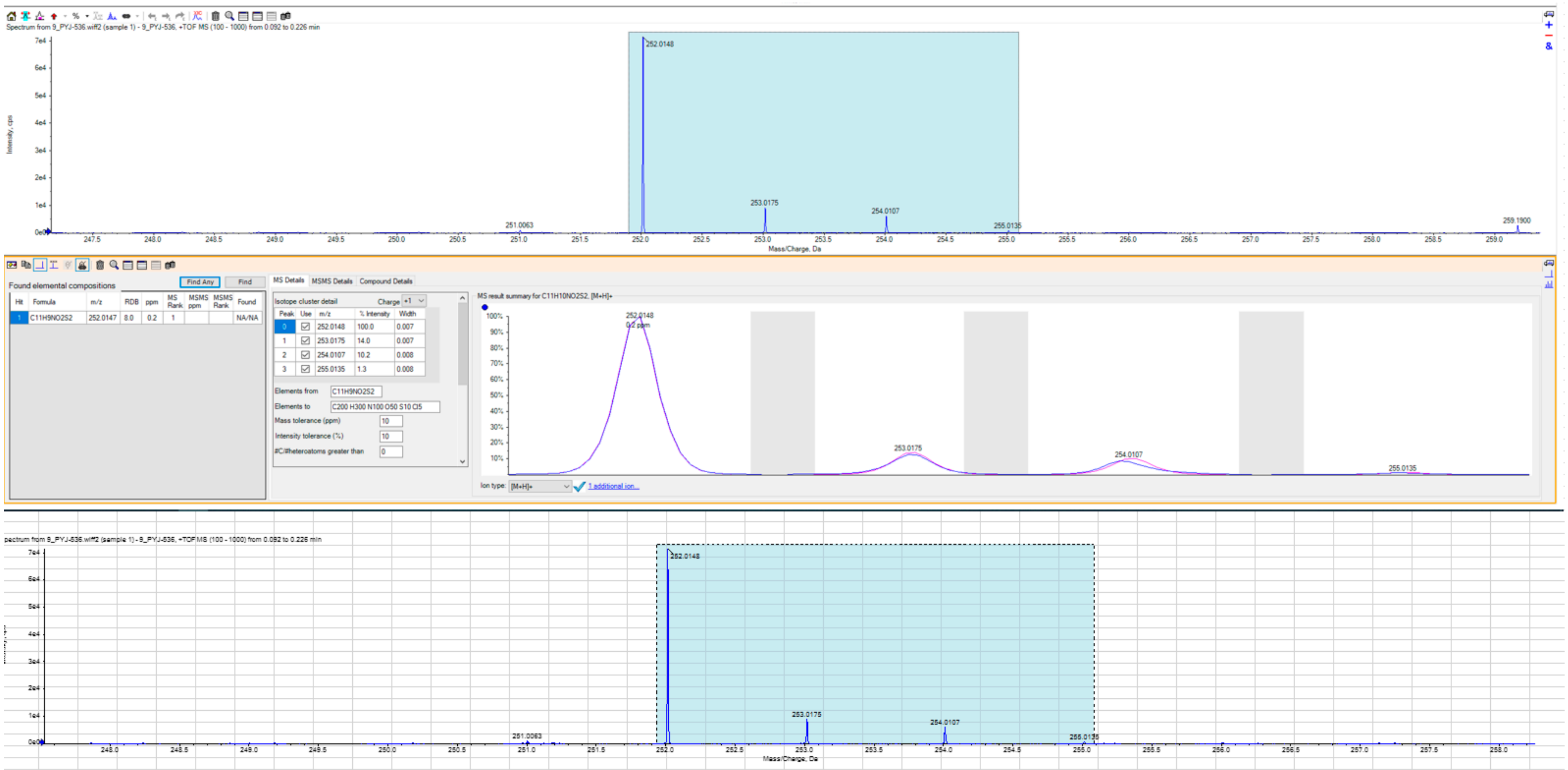

Figure S17. HRMS spectrum of analog **1**

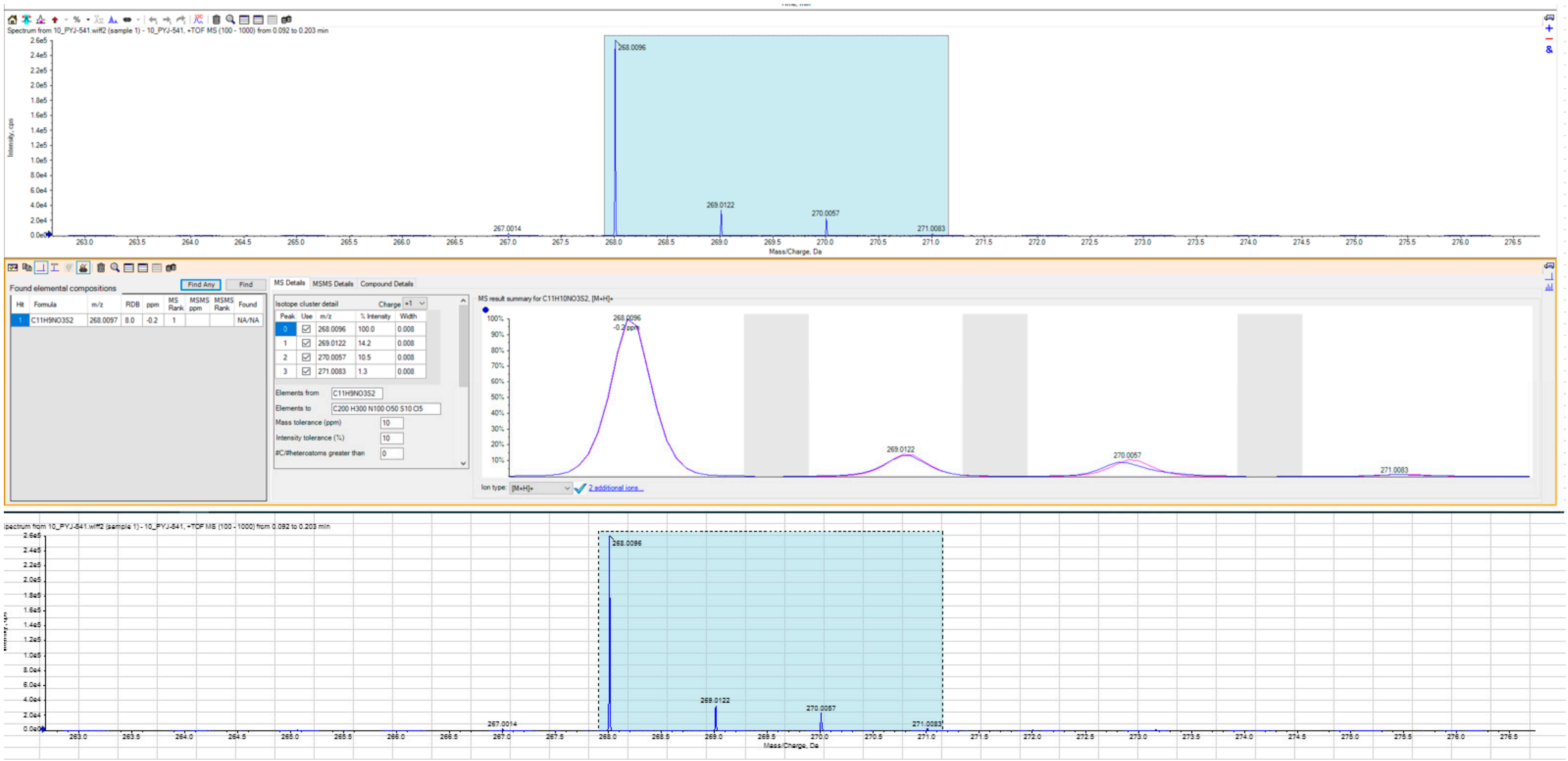

Figure S18. HRMS spectrum of analog **3**

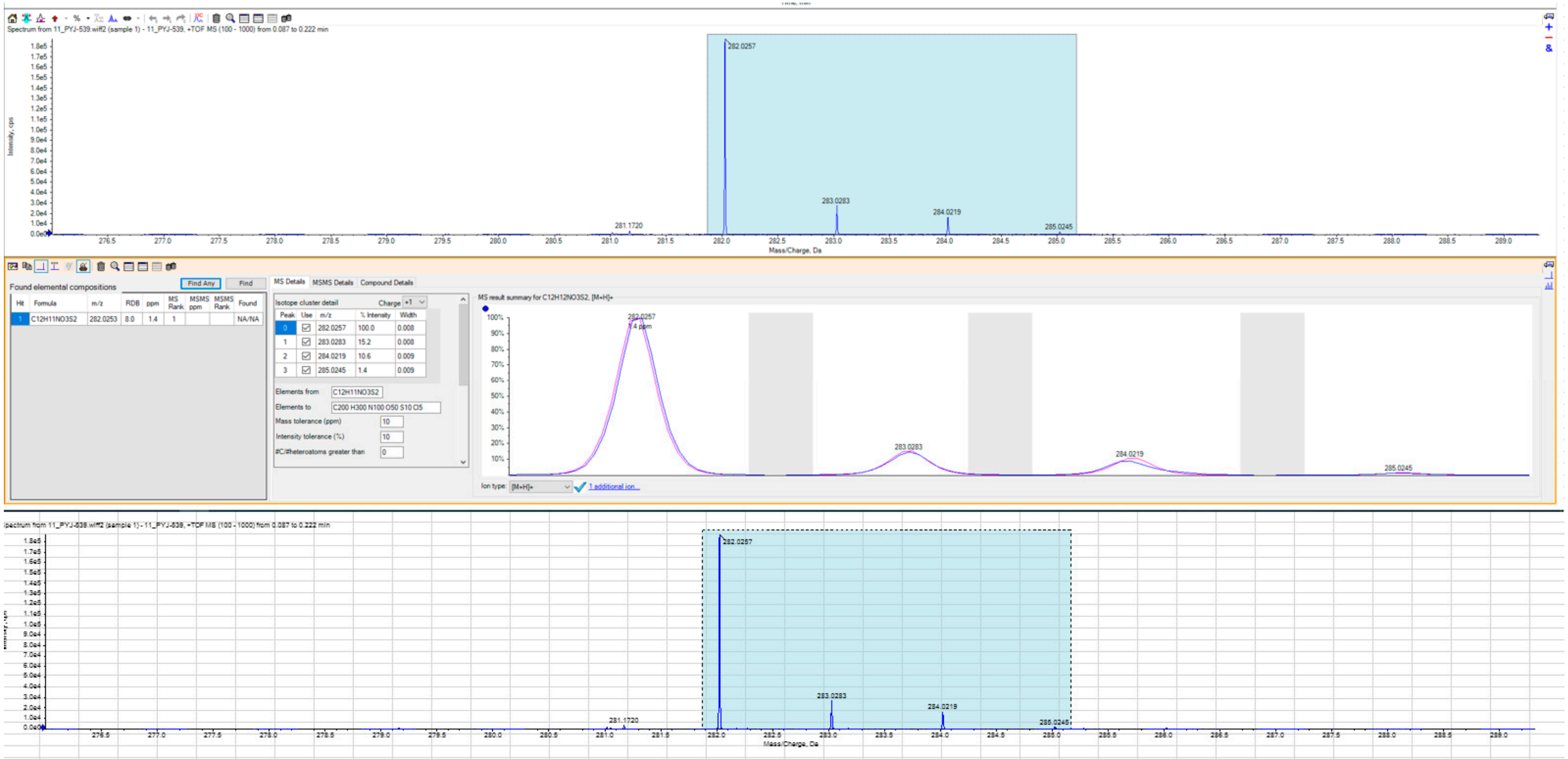

Figure S19. HRMS spectrum of analog **5**
